# Callose deposition analysis with special emphasis on plasmodesmata ultrastructure during megasporogenesis in *Sedum* (Crassulaceae)

**DOI:** 10.1007/s00709-023-01879-x

**Published:** 2023-07-07

**Authors:** Emilia Brzezicka, Małgorzata Kozieradzka-Kiszkurno

**Affiliations:** Department of Plant Cytology and Embryology, Faculty of Biology, University of Gdańsk, 59 Wita Stwosza St., 80-308 Gdańsk, Poland

**Keywords:** Cell wall, Monosporic megasporogenesis, Ovule, Plant reproductive development, Ultrastructure

## Abstract

In this study, the results of the first detection of callose within the ovules of the representatives of the family Crassulaceae are presented. This study was carried out on three species of the genus *Sedum*. Data analysis showed differences in the callose deposition pattern between *Sedum hispanicum* and *Sedum* ser. *Rupestria* species during megasporogenesis*.* Callose was present mostly in the transversal walls of dyads and tetrads in *S. hispanicum.* Furthermore, a complete loss of callose from the cell walls of the linear tetrad and a gradual and simultaneous deposition of callose within the nucellus of *S. hispanicum* were observed. The findings of this study showed the presence of hypostase with callose in the ovules of *S. hispanicum*, which is not common in other angiosperms. The remaining species tested in this study—*Sedum sediforme* and *Sedum rupestre*—showed a typical, well-known callose deposition pattern for plants with the monospore type of megasporogenesis and the Polygonum type of embryo sac. The functional megaspore (FM) in all studied species was located most chalazally. FM is a mononuclear cell, which wall is callose-free in the chalazal pole. The study presents the causes of different patterns of callose deposition within *Sedum* and their relationship with the systematic position of the study species. Moreover, embryological studies present an argument for excluding callose as a substance that forms an electron-dense material near the plasmodesmata in megaspores of *S. hispanicum*. This research expands the knowledge about the embryological processes of succulent plants from the family Crassulaceae.

## Introduction

Megasporogenesis and megagametogenesis are key processes that demonstrate the course of the development of the megaspores and the female gametophyte, respectively. Megasporogenesis results in the formation of a functional megaspore (FM), which then develops into a megagametophyte during megagametogenesis. Chemical changes, i.e., the deposition of callose (β-1,3-glucan), take place in the walls of the megasporocyte and the cells dividing during megasporogenesis. The presence of callose is reported in plants with the monosporic and bisporic types of megasporogenesis, in which, finally, the active megaspore (embryo sac mother cell) is mononuclear or binuclear, respectively. Callose deposition is not observed in plants with the tetrasporic type of megasporogenesis, in which all four nuclei formed after meiosis are part of the FM (Rodkiewicz and Górska-Brylass 1968; Rodkiewicz 1970; Bhandari 1984; Reiser and Fischer 1993; Lee and Yeung 2012; Hojsgaard and Pullaiah 2022). Callose distribution in the walls of the abovementioned cells is not accidental and undergoes dynamic changes as a result of callose production by callose synthases and callose digestion probably by β-1,3-glucanases. However, the biochemical mechanism of callose synthesis and degradation has not yet been precisely described (Chen and Kim 2009).

The placement of the FM with respect to the micropylar–chalazal axis is commonly reflected in the pattern of callose deposition. This is particularly evident in plants with, for example, Polygonum and Oenothera types of embryo sacs, which was comprehensively presented for 14 angiosperm families by Rodkiewicz (1970, 1973). Generally, the location of the first manifestations of callose deposition in the megaspore mother cell (MMC) wall and its disappearance during later steps of megasporogenesis (dyad or tetrad stage) reflect the position of the FM placement in the tetrad (Rodkiewicz and Górska-Brylass 1968; Rodkiewicz 1970; Kuran 1972; Bouman 1984). However, the apostasy of the above rule related to the first manifestations of callose accumulation in the megasporocyte and the location of the FM was also described, for example, in a monocot plant with the Polygonum type of embryo sac—*Arundo formosana* (Poaceae) (Jane and Chiang 1996). Nevertheless, the relationship between callose distribution and dissolution and the selection of the FM is observed in angiosperms (Rodkiewicz 1970; Tucker and Koltunow 2014), e.g., *A. formosana* and also diplosporic apomicts from the family Asteraceae (Musiał et al. 2015; Musiał and Kościńska-Pająk 2017; Musiał and Kościńska-Pająk 2019). The role of callose in megasporogenesis and in the selection of the FM has not been fully understood (Tucker and Koltunow 2009; Chen and Kim 2009; Drews and Koltunow 2011; Hojsgaard and Pullaiah 2022). An in-depth understanding of the function of callose in reproductive development can be achieved by analyzing the callose deposition pattern in more species, including sexual and apomictic ones (Janas et al. 2022). The primary aim of the present study is to investigate, for the first time, the pattern of callose deposition in species of the family Crassulaceae. This aim is particularly important due to the current, unique observations on the ultrastructure of developing cells during megasporogenesis, megagametogenesis, and embryogenesis of the family Crassulaceae—the presence of plasmodesmata with an adjacent electron-dense material. Many roles of callose in plant development and growth—FM selection, cell plate formation, and plasmodesmata regulations (Chen and Kim 2009)—are crucial for the study and analysis of megasporogenesis and also in the context of the present research.

Although the embryological research of the species of the family Crassulaceae was carried out in the nineteenth century using light microscopy (Rombach 1911 and literature therein; Sharp 1913; Mauritzon 1933 and literature therein), callose detection has not been performed in the ovules of Crassulaceae until now. However, ultrastructural analyses of ovules in some species of Crassulaceae concluded that plasmodesmata with an adjacent electron-dense material are a unique feature for all Crassulaceae species studied so far (Kozieradzka-Kiszkurno and Bohdanowicz 2010; Kozieradzka-Kiszkurno et al. 2012; Kozieradzka-Kiszkurno et al. 2020; Brzezicka and Kozieradzka-Kiszkurno 2021). This feature makes the representatives of Crassulaceae an interesting subject for further research in embryology, which has the potential to provide new, important data on intercellular connections in angiosperms.

The species selected for this study belong to the third largest family of succulent plants—Crassulaceae (about 1400 species)—specifically, the largest, highly polyphyletic genus of the family—*Sedum* (about 420 species) (Nikulin et al. 2016; Smith et al. 2019). Since the available embryological data on Crassulaceae species are significantly limited, this study aimed to detect callose during megasporogenesis; describe the potential function of callose and observe dynamic changes in callose deposition in three species of the genus *Sedum* (*S. hispanicum* L., *S. sediforme* (Jacq.) Pau., and *S. rupestre* L.), which are the comprehensively (cytochemically and ultrastructurally) studied species so far; and compare the callose distribution pattern. Among the three species studied, *S. sediforme* and *S. rupestre* belong to *Sedum* ser. *Rupestria*. This monophyletic group of plants is currently recognized to the genus level—*Petrosedum* Grulich (Thiede and Eggli 2007; Gallo and Zika 2014; Nikulin et al. 2016; Messerschmid et al. 2020). To the best of our knowledge, simultaneous anatomical, cytochemical, and ultrastructural analyses of the cells during megasporogenesis and the development of the female gametophyte were carried out only in the three aforementioned Crassulaceae species (Brzezicka and Kozieradzka-Kiszkurno 2018, 2019, 2021). The present study tested the hypothesis that the pattern of callose deposition differs in the studied plants, which is related to the systematic position of the species.

The embryological data obtained during the research were compared with the results obtained so far for *Sedum*, which made precise comparative analysis possible. The additional ultrastructural analysis of the plasmodesmata in *S. hispanicum*—the only species showing the presence of plasmodesmata with electron-dense material during megasporogenesis—allowed us to draw reliable conclusions. The chemical characteristics of the material visible near the Crassulaceae plasmodesmata are not yet determined (Wróbel-Marek et al. 2017). The present study aimed at verifying the hypothesis that there is a relationship between the pattern of callose deposition and the occurrence of plasmodesmata with an electron-dense material in *Sedum*. The results obtained were discussed in relation to the possible roles of callose in Crassulaceae species during megasporogenesis and female gametophyte development in light of the available embryological data for other angiosperms.

## Materials and methods

### Plant material

Ovules of *S. hispanicum*, *S. sediforme*, and *S. rupestre* were isolated from flower buds of various sizes at different stages of development for microscopic analysis. Megasporogenesis was studied at the individual stages, i.e., at the stage of megaspore mother cell (MMC), dyad (Dy) and triad (Tr)/ tetrad (Te) development. Megagametogenesis analysis included the formation of cellular female gametophyte (ES). Callose was assessed in 258 ovules of *S. hispanicum* (i.e., MMC: 55/Dy: 46/Te: 103/ES: 54), 206 ovules of *S. sediforme* (i.e., MMC: 52/Dy: 43/Tr: 89/ES: 22), and 243 of *S. rupestre* (i.e., MMC: 61/Dy: 55/Tr: 94/ES: 33). The data obtained during experiment are consistent for each species across all samples.

Plants of *S. sediforme* were obtained from a collection from the Botanical Garden of the Jagiellonian University in Cracow (Poland). Plant materials of *S. hispanicum* and *S. rupestre* were collected from a natural habitat in Gdańsk in northern Poland. The flowers used in the isolation of ovules were harvested during the growing seasons in the years 2017–2020.

### Ovule clearing technique and callose detection

Ovules were analyzed at successive stages of megasporogenesis using a light microscope with Nomarski interference contrast optics and a fluorescence microscope. Callose detection was carried out using decolorized aniline blue (DAB, Polyscience C.I. 42755), following the procedures of Rojek et al. (2018) and Hedhly et al. (2018). The samples were incubated in 1% SDS (sodium dodecyl sulfate) and 0.2 N NaOH solutions at room temperature and washed in distilled water. The plant materials were stained overnight with 0.05% DAB in 0.1 m KH_3_PO_4_. The ovules were studied on a microscope slide using a Nikon Eclipse E 800 microscope with an Epi-Fl Filter Block N UV-2A (EX 330–380, DM 400, BA 435–485) under UV light for callose observations and using a differential interference contrast optic for anatomical observations of cleared specimens.

### Transmission electron microscopy (TEM) and semithin section observations

For ultrastructural analysis, the plant materials were fixed with 2.5% glutaraldehyde and 2.5% formaldehyde (from paraformaldehyde) in 0.05 M cacodylate buffer (pH 7.0) at room temperature (4 h). Then, the ovules washed with the cacodylate buffer were rinsed with 1% osmium tetroxide overnight at 4°C. After dehydration in serial acetone solutions, the ovules were embedded in Spurr’s epoxy resin (Spurr 1969) and cut on a Leica EM UC7 ultramicrotome according to the previously used procedures of Crassulaceae embryological studies (Kozieradzka-Kiszkurno and Bohdanowicz 2010). Observations were made using an FEI Tecnai G2 Spirit TWIN/BioTWIN transmission electron microscope at 120 kV at the Laboratory of Electron Microscopy/Faculty of Biology/University of Gdańsk (Poland). Semithin control sections were stained with 0.05% toluidine blue O and observed using a Nikon Eclipse E 800 light microscope.

## Results

### Callose deposition pattern during megasporogenesis in *Sedum* ser. *Rupestria*

**Fig. 1:**
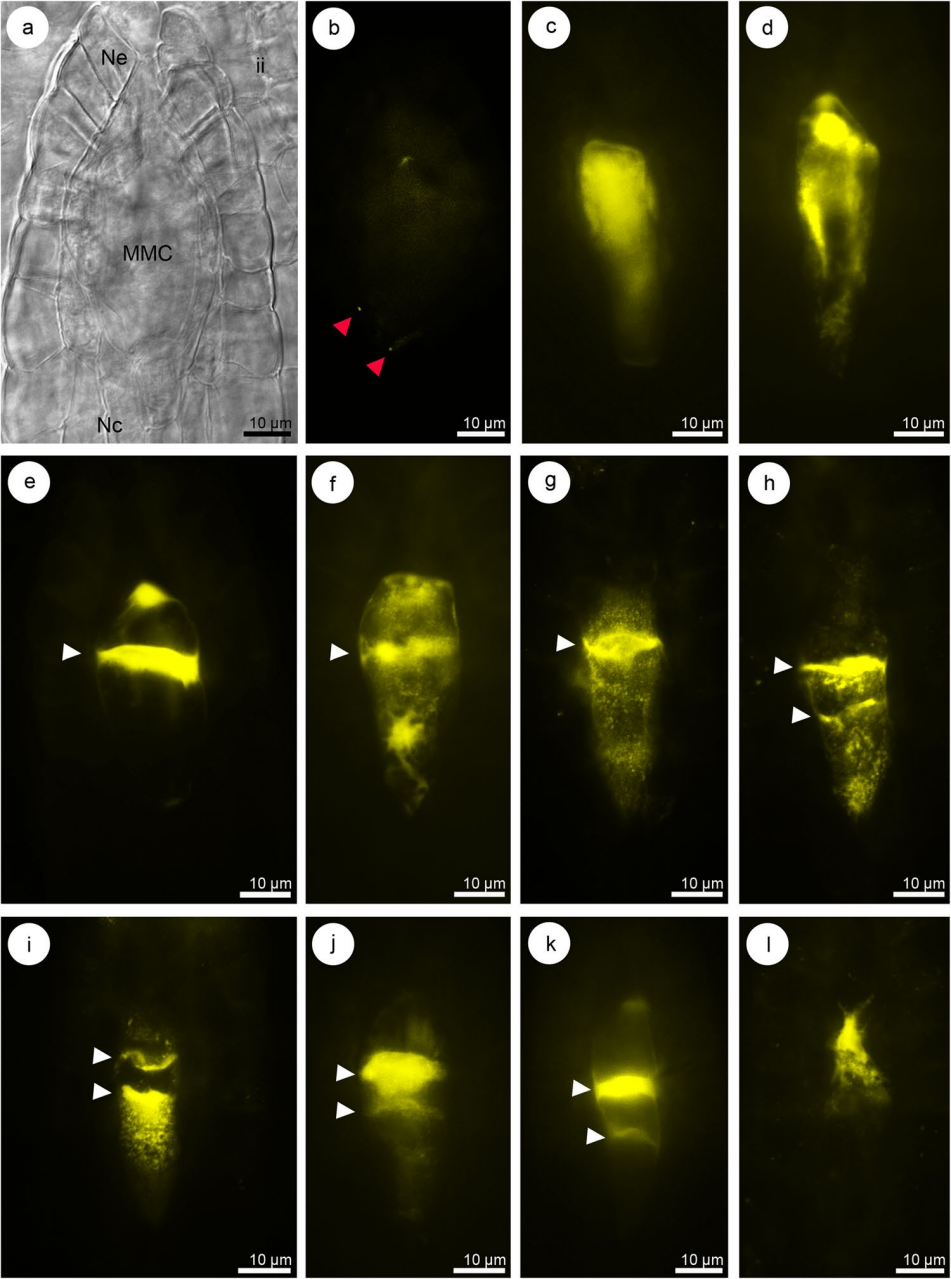
Megasporogenesis and callose distribution in the ovules of *Sedum* ser. *Rupestria* (**a**–**f**, **j**–**k**: *Sedum sediforme*; **g**–**i**, **l**: *Sedum rupestre*). **a** Nomarski differential interference contrast optic, **b**–**l** aniline blue staining results (yellow). Transversal wall formation with callose deposition is represented by white arrowhead. The micropylar pole is present at the top of all photographs. **a** Megaspore mother cell (MMC) located within the bitegmic ovule: inner integument (ii), nucellar cells (Nc), nucellar epidermis (Ne). **b** Callose deposition visible on the chalazal side of the meiocyte (red arrowheads). **c**, **d** MMC stage with callose accumulation visible around the cell. Callose presence in the transversal (**e**–**g**) and side walls (**f**, **g**) of the dyad. **h**–**k** Triad stage with a visible callose presence in the transversal walls. Callose accumulation is decreased in the chalazal pole of the triad/chalazal megaspore. **i** Callose accumulation around nonfunctional cells. Functional megaspore is callose-free at the chalazal side

The results obtained for the *Sedum* ser. *Rupestria* species (*S. sediforme* and *S. rupestre*) were similar; therefore, they were presented together. Initially, callose accumulation was not observed in the archesporial cell that forms in the young ovule. The first signs of the presence of callose were observed locally in MMC walls in the chalazal pole of the cell. The fluorescence of callose was detected at the prophase I stage of the meiocyte (Fig. 1a, b). In the later stages of nuclear division, callose deposition was increased so that intensive fluorescence extended over almost the entire cell (Fig. 1c, d). The nuclei formed as a result of the first meiotic division were located in two different cells after cytokinesis. A strong fluorescence and callose abundance were observed in the transverse walls, which separate the dyad cells (Fig. 1e–g). Dynamic changes in callose deposition during megasporogenesis led to an increase in callose deposition in sidewalls (Fig. 1f, g). The division of the chalazal dyad cell resulted in a linearly arranged triad of cells with callose deposition on the transverse and sidewalls (Fig. 1h–k). In general, the FM that gives rise to the female gametophyte in the tested species of *Sedum* is a mononuclear cell located chalazally and surrounded by callose walls partially, and it is ultimately (on the chalazal side) callose-free (Fig. 1l). The decrease in callose fluorescence was first observed in the chalazal pole of the triad—specifically, in the chalazal megaspore (Fig. 1i, k). This observation made in *Sedum* shows the relationship between the disappearance of callose and the location of the embryo sac mother cell in the triad. Compared with the surrounding ovular cells, the germline cells were clearly distinguished by the occurrence of callose.

### Callose detection in the ovules of *Sedum hispanicum* and TEM analysis

**Fig. 2:**
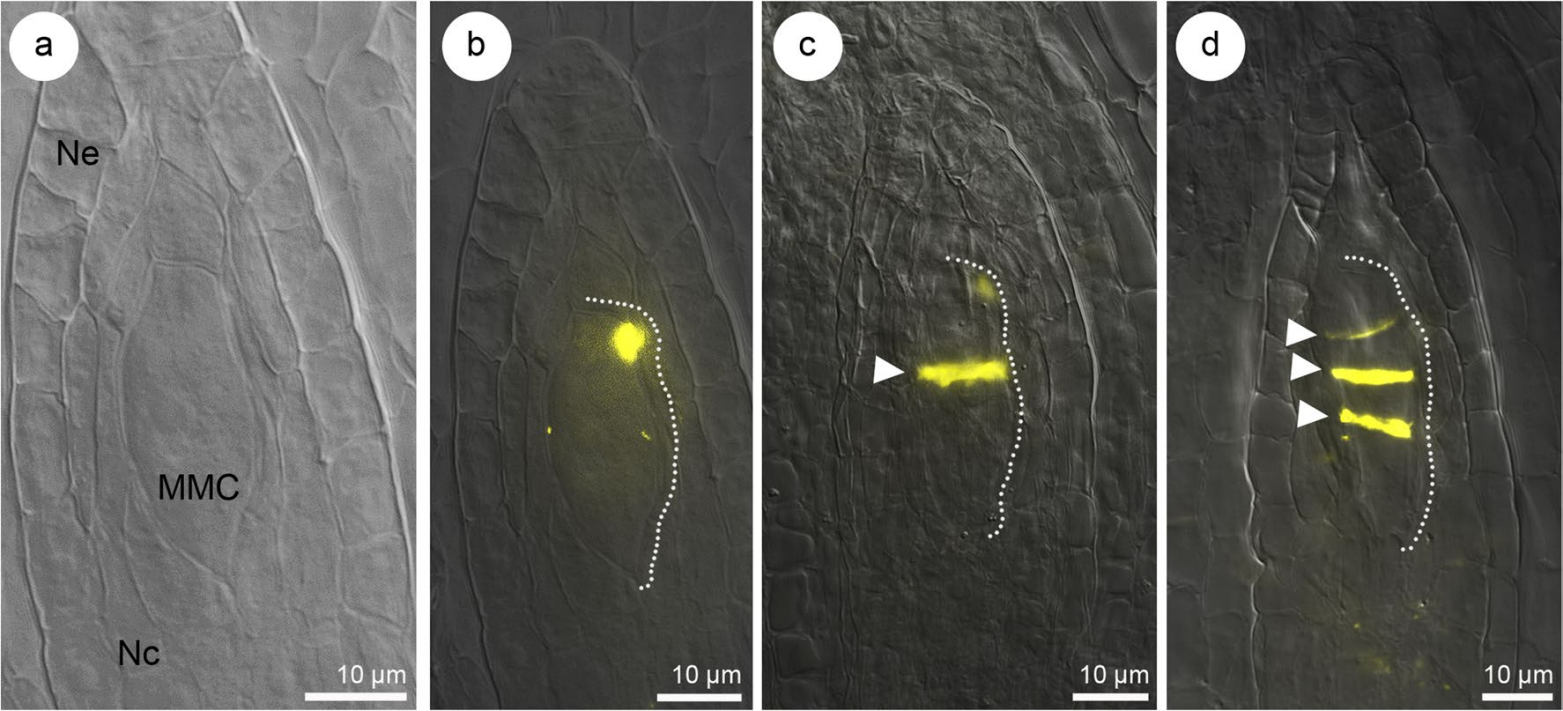
Callose accumulation pattern during megasporogenesis of *Sedum hispanicum*. **a** Nomarski differential interference contrast (DIC) optic, **b**–**d** merged images obtained using Nomarski DIC optics with aniline blue staining results (yellow). Transversal wall formation with callose deposition is represented by white arrowhead. The micropylar pole is present at the top of all photographs. **a** Megaspore mother cell (MMC) within the nucellus of the ovule. **b** Callose accumulation at the megaspore mother cell stage. **c** Dyad stage with visible callose-free side walls. **d** Callose accumulation in the transverse walls of the tetrad. Nucellar cells (Nc), nucellar epidermis (Ne)

**Fig. 3:**
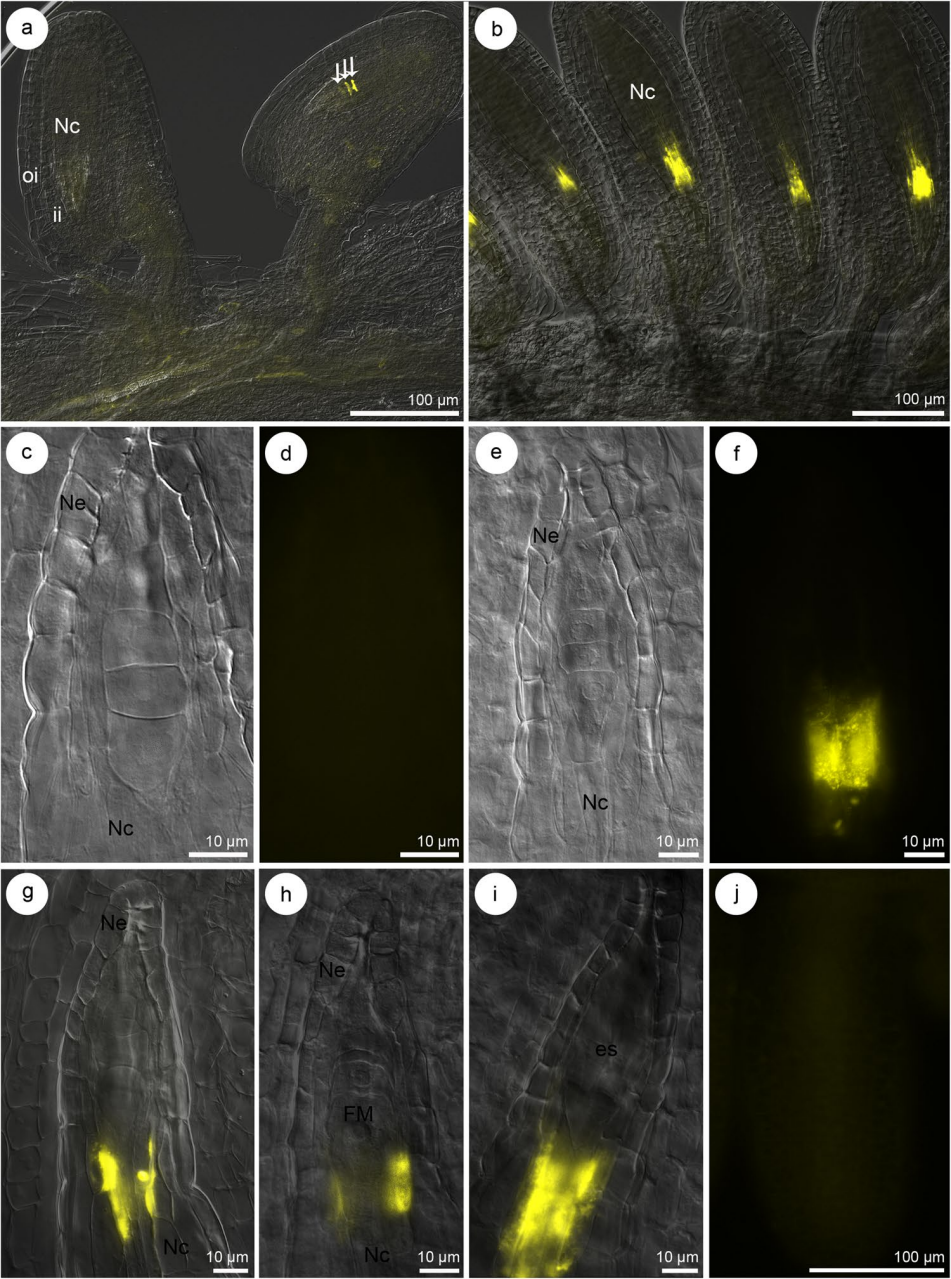
Callose distribution in the ovule of *Sedum hispanicum* during the tetrad stage, functional megaspore (FM), and embryo sac formation. **a**, **b**, **g**–**i** Merged images obtained using Nomarski differential interference contrast (DIC) optics with aniline blue staining results (yellow), **c**, **e** Nomarski DIC optic, **d**, **f** aniline blue staining results (yellow), **j** negative control. The micropylar pole is present at the top of the photographs (**c**–**j**). **a** Callose accumulation in the transverse walls of the tetrad (white arrows). **b** Ovules with a visible fluorescent hypostasis. **c**, **d** Tetrad stage with a lack of callose accumulation (**d**). **e**–**g** Tetrad stage with a visible callose presence in hypostasis cell walls (**f**, **g**). **h** Fluorescent hypostasis occurrence during FM formation. **i** Cellular embryo sac stage with adjacent fluorescent hypostasis at the chalazal pole of the megagametophyte. **j** The image of a negative control shows the ovule of *S. hispanicum*. Embryo sac (es), inner integument (ii), nucellar cells (Nc), nucellar epidermis (Ne), outer integument (oi)

**Fig. 4:**
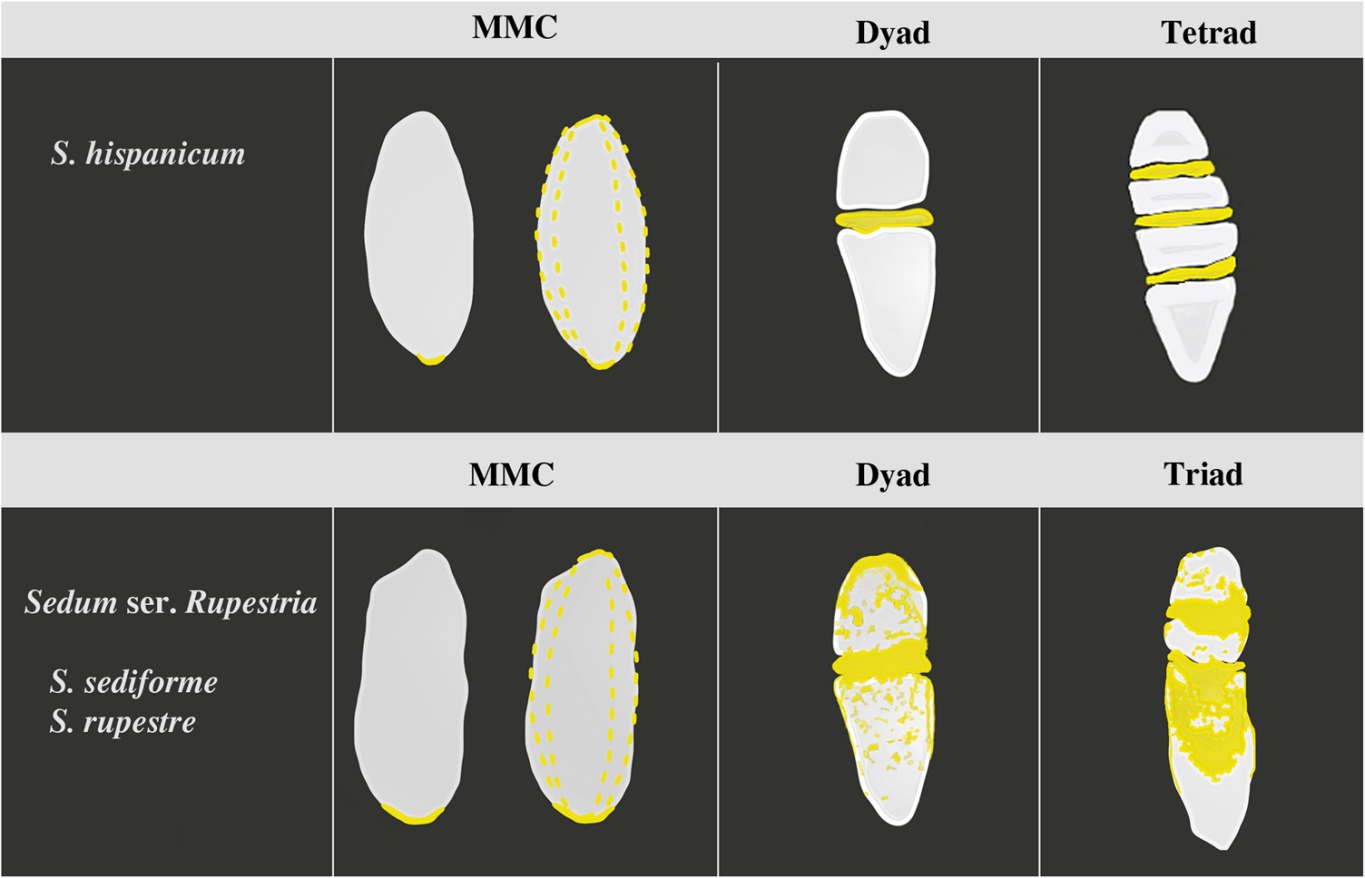
Summary scheme of the dynamic of callose accumulation over time in *S. hispanicum* and *Sedum* ser. *Rupestria* ovules during representative and subsequent stages of megasporogenesis*.* Data are presented together for *S. sediforme* and *S. rupestre* due to significant similarity; *S. hispanicum*—(MMC stage) callose occurs in the chalazal pole of the cell or it is present punctate in the whole cell wall (the dotted line); (dyad and tetrad stage) callose deposition is primarily visible in the transverse walls; *Sedum* ser. *Rupestria* (*S. sediforme* and *S. rupestre*)—(MMC stage) callose occurs in the chalazal pole of the cell or it is extended over almost the entire cell (the dotted line); (dyad and triad stage) transverse and side walls of the dyad and a linearly arranged triad of cells are clearly rich in callose. The callose plates located between the cells are visibly thick. Chalazally located cells of the dyad and triad (mononuclear functional megaspore) are characterized by relatively less callose deposition at the chalazal pole than in other parts of the cell

Callose fluorescence was observed in meiocyte walls (Fig. 2a, b). The dyad, which was formed as a result of the first meiotic division after cytokinesis, showed a weaker callose fluorescence in sidewalls. A more intense callose staining was observed in transverse walls, separating the dyad cells (Fig. 2c). Megasporogenesis resulted in a tetrad of megaspores, which transverse walls were primarily characterized by callose deposition (Figs. 2d, 3a, and 4). The cell wall, separating the megaspore located the closest to the micropyle, showed weaker fluorescence than the others in the tetrad (Figs. 2d and 3a).

**Fig. 5:**
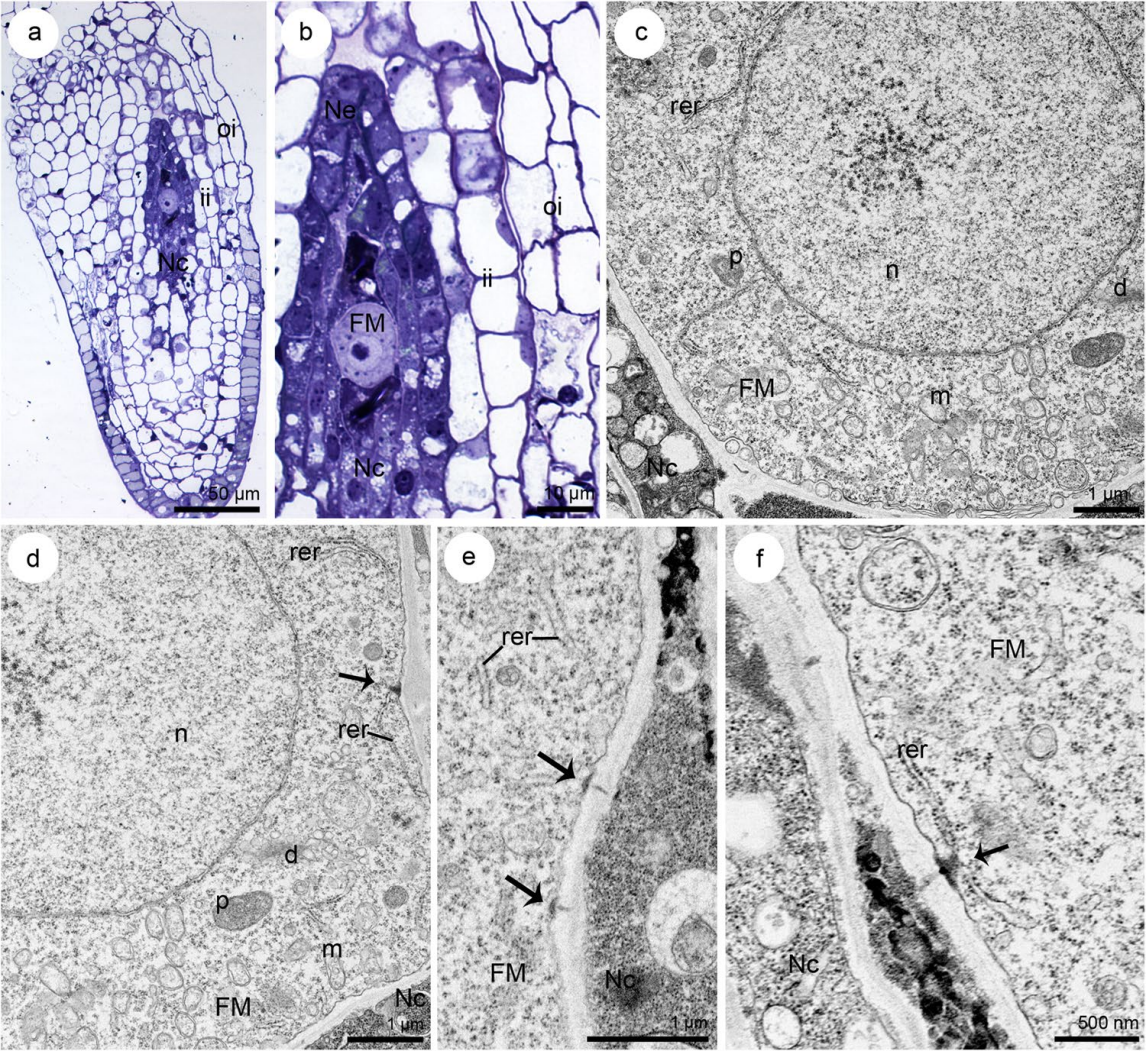
The structure of the functional megaspore (FM) in *Sedum hispanicum*. **a**, **b** Light microscope, **c**–**f** transmission electron microscope. **a** Anatropous, bitegmic ovule at the FM stage. **b** FM and ovular tissues from (**a**) visible under high magnification. **c**, **d** Chalazal pole of the uninucleate (n) FM from (**b**) with visible plastids (p), mitochondria (m), single profiles of rough endoplasmic reticulum (rer), and dictyosomes (d) in the cytoplasm. Plasmodesmata with electron-dense material occur in the cell walls (arrow). **e**, **f** Profiles of rough endoplasmic reticulum (rer) occur near the plasmodesmata (arrow) and come into contact with the electron-dense material (**f**) from the FM side. Inner integument (ii), nucellar cells (Nc), nucellar epidermis (Ne), outer integument (oi)

Dynamic changes were observed in the callose distribution pattern during the development of megaspores. The detection of callose at the tetrad stage in *S. hispanicum* revealed megaspores in which callose was no longer visible (Fig. 3c, d)—42 ovules (including 16 ovules without callose presence in the walls of the tetrad and nucellus, 26 ovules without callose presence in the walls of tetrad, but with its presence in the nucellus). The lack of callose detection in tetrads of *S. hispanicum* was consistently observed during the ovule development (Fig. 3c, d) and it was related, with the gradual and simultaneous deposition of callose in the walls of the nucellus (Fig. 3b, e–g). Intensive fluorescence was observed in the nucellar cells located near the chalazal megaspore. Callose accumulation was observed even before the degeneration of non-FMs within the cell walls of the nucellar cells (Fig. 3h). The pronounced fluorescence of the neighboring nucellar cells was maintained during megagametogenesis until the cellular embryo sac stage (Fig. 3b, i). The fluorescence was not observed within the nucellus (457 ovules analyzed) of *S. hispanicum* in a negative control, where aniline blue staining was omitted (Fig. 3j). Structural analysis of FMs conducted after degeneration of non-FMs in *S. hispanicum* (Fig. 5a, b) revealed the presence of mitochondria, single profiles of rough endoplasmic reticulum (RER), plastids, and active dictyosomes in the cytoplasm (Fig. 5c, d). The cell walls of the FM were not visibly thickened. In addition, simple plasmodesmata with adjacent electron-dense material from the cytoplasm side of the FM were observed. The profiles of RER were located near the electron-dense dome (Fig. 5d–f).

## Discussion

The results of the present study are consistent with the embryological observations made so far in *S. hispanicum*, *S. sediforme*, and *S. rupestre* during megasporogenesis *(*Mauritzon 1933; Brzezicka and Kozieradzka-Kiszkurno 2018, 2019, 2021). Megasporogenesis resulted in a triad of cells in *S. sediforme* and *S. rupestre*, whereas in *S. hispanicum*, a tetrad was produced (Brzezicka and Kozieradzka-Kiszkurno 2018, 2019, 2021). The present study showed that chemical changes (deposition of callose) occur in the walls of the meiocyte and the cells that formed during the monospore type of megasporogenesis. However, the callose deposition pattern was different in the tested *Sedum* species (Fig. 4). Despite the differences in the callose deposition pattern between *S. hispanicum* and the species of *Sedum* ser. *Rupestria*, the callose distribution pattern in *Sedum* is similar to that presented and commonly known in angiosperms with the monospore type of megasporogenesis and the Polygonum type of embryo sac.

The first signs of the callose appearance in *Sedum* ovules were observed in the walls of the megasporocyte at the first meiotic division—prophase stage. In *Sedum*, callose allows to identify cells that enter megasporogenesis and meiotic division. The MMC of the studied *Sedum* species is the only cell within the micropylar part of the nucellus that initiates the formation of FM and shows callose deposition. These findings indicate that callose is a histological marker of MMC and that it allows to recognize young reproductive cells in the ovules of *Sedum*, similar to the findings reported in other angiosperms (Drews and Koltunow 2011; Tucker and Koltunow 2014)*.*

The function of callose during the development of the female gametophyte has not been precisely specified, but its possible role in FM selection, regulation of intercellular communication, isolation of generative cells from sporophyte tissues, and formation of some kind of temporal filter, which can be an additional source of signaling molecules, has been hypothesized (Bouman 1984 and literature therein; Ünal et al. 2013 and literature therein; Tucker and Koltunow 2009, 2014 and literature therein). Callose is a molecular and nutritional filter that contributes to the decrease in the permeability of the cell walls, especially for larger molecules. Temporal and selective isolation of generative cells from sporophyte tissues allowing them begins an independent development path (Heslop-Harrison and Mackenzie 1967; Rodkiewicz and Górska-Brylass 1968; Rodkiewicz 1970; Bouman 1984). This isolation is associated with the transition from sporophytic to gametophytic gene expression (Musiał et al. 2015) and the acquisition of a generative identity by cells. However, the function of callose during reproductive processes has not yet been precisely described, which requires a deeper understanding (Tucker and Koltunow 2009; Chen and Kim 2009; Drews and Koltunow 2011 and literature therein). In the studied *Sedum* species, the chalazal megaspore that develops into the embryo sac during megagametogenesis was finally callose-free. Only weak callose fluorescence was detected in the micropylar region of the FM and in nonfunctional ones in *Sedum* ser. *Rupestria*, which are—before, during, and after degeneration—similar to other Angiospermae (Rodkiewicz and Górska-Brylass 1968; Rodkiewicz 1970). This shows that the callose deposition pattern is related to the placement of the FM in tetrads of *S. hispanicum* and triads of *S. sediforme* and *S. rupestre*. The findings of the present study are consistent with those of previous studies and support the possibility of the role of callose in the above-described processes in *Sedum.*

In *S. hispanicum*, intensive fluorescence of callose was observed in the tissues of the ovules. Callose was observed in the walls of the nucellar cells, which were located near the chalazal side of the megaspores, coenocytic and later cellular embryo sac. This nucellar tissue can differentiate in hypostase, which structure is variable. One of the many structural elements of hypostase is the impregnation of its cell walls with callose (Maheshwari 1950; Tilton 1980; Bouman 1984). This is consistent with the observations of the present study. Callose has also been observed in the walls of the hypostasis cells of other Angiosperms (Rodkiewicz 1970; Bouman 1984 and literature therein), e.g., *Antirrhinum majus* (Rodkiewicz 1967) and some representatives of the family Scrophulariaceae (Kuran 1972). Fluorescent hypostasis in species from the family Scrophulariaceae, e.g., *Digitalis lanata*, *Digitalis sibirica*, *Digitalis purpurea*, and *Pentstemon aureus*, masks the chalazal pole of the megasporocyte and the formation of cells up to the tetrad stage. The degree of the callose deposition in longitudinal walls of the cells formed during megasporogenesis in the representatives of the family Scrophulariaceae differs regardless of the presence of fluorescent hypostasis (Kuran 1972). This shows that the occurrence of hypostase is specific to the species and does not exert a crucial influence on the callose deposition pattern during the formation of megaspores. Species showing fluorescence only in the transverse walls of the dyad and tetrad (*P. aureus*) and those in which the presence of callose is also observed in longitudinal cell walls (*D. lanata, D. sibirica, D. purpurea*) show simultaneously the presence of fluorescent hypostasis in the ovules. The feature that distinguishes the aforementioned species from other species of the family Scrophulariaceae with the Polygonum type of embryo sac is the lack of callose fluorescence in the chalazal pole of the megasporocyte. This is most likely due to the masking of the chalazal part of the MMC by hypostase, which encircles them. This observation is confirmed by the observations in *Digitalis ambigua*, which also has hypostasis, but its fluorescence occurs later so that fluorescence at the chalazal pole of the megasporocyte is visible (Kuran 1972). Moreover, this observation is also confirmed by the findings of the present study in *S. hispanicum* ovules. In *S. hispanicum*, fluorescent hypostasis appears at the tetrad stage, which also enables observation of callose fluorescence on the chalazal side of the megasporocyte.

The function of hypostase has not yet been precisely understood. Hypostase may be built of metabolically active cells involved in the stopping of the transport of substances to the embryo sac, or on the contrary, it may facilitate the transport. It also plays a protective function and produces some hormones (Maheshwari 1950; Tilton 1980; Bouman 1984; Pullaiah et al. 2019; Raghavan 2000; Thijssen et al. 2003). Due to the multifunctionality of hypostase, it is difficult to unequivocally indicate the role of hypostasis in *S. hispanicum*. However, based on the results obtained for *Sedum* species and the available literature data, the discussion of the protective function of hypostasis seems to be most important, which is built of thick-walled cells. The intense elongation of the female gametophyte toward the chalazal pole was observed during megagametogenesis in species of *Sedum* ser. *Rupestria*, whereas this tendency was not observed in *S. hispanicum* (Brzezicka and Kozieradzka-Kiszkurno 2018, 2019). These findings indicate the possibility of the formation of some kind of barrier tissue by hypostasis, which is involved in the limitation of the elongation and encroachment of the embryo sac in *S. hispanicum* ovules*.*

The callose deposition pattern may vary depending on the type of megasporogenesis and the species (Rodkiewicz 1970; Kuran 1972). Moreover, the callose accumulation pattern during megasporogenesis is a characteristic feature of the species. This observation is consistent with the observations of the present study of three Crassulaceae species. However, the available embryological data on the representatives of the family Orchidaceae demonstrate the specific distribution of callose (Kuran 1972, Bouman 1984 and literature therein). Differences in the callose deposition pattern between *S. hispanicum* and the *Sedum* ser. *Rupestria* species were associated with the weaker callose fluorescence in the sidewalls of *S. hispanicum*, callose accumulation primarily in the transverse walls of the dyad and tetrad, and also the presence of hypostase with callose material. Moreover, the complete and relatively early disappearance of callose from the walls of the tetrad was observed in *S. hispanicum*, even before the complete degeneration of non-FMs. The different observation in the *Sedum* ser. *Rupestria* species compared with *S. hispanicum* seems to be another, though quite discreet, cytoembriological feature with a little specificity in relation to other Angiospermae, which distinguishes them from the representatives of the genus *Sedum* studied so far. These observations constitute an additional argument in favor of separating *Sedum* ser. *Rupestria* and giving it the rank of the genus *Petrosedum*. These findings are consistent with the descriptions made so far during morphological (Thiede and Eggli 2007 and literature therein; Giuliani et al. 2018), molecular (Nikulin et al. 2016; Messerschmid et al. 2020), and embryological analyses (Mauritzon 1933). Embryological data support their distinct nature, such as the *Rupestre* type of the endosperm (Mauritzon 1933), the filamentous structure of embryo suspensor (Mauritzon 1933; Kozieradzka-Kiszkurno et al. 2020), the absence of the characteristic feature for Crassulaceae plasmodesmata in the walls of the suspensor basal cell (Czaplejewicz and Kozieradzka-Kiszkurno 2013; Kozieradzka-Kiszkurno et al. 2020), the occurrence of the triad as a result of megasporogenesis (Brzezicka and Kozieradzka-Kiszkurno 2019, 2021), the formation of plasmodesmata with electron-dense material in the outer walls of antipodal cells (Brzezicka and Kozieradzka-Kiszkurno 2019, 2021), and the absence of hypostasis with callose. However, further research is needed to verify whether the presence of hypostasis with callose material is common for all species of the genus *Sedum* or it is a species-dependent characteristic.

Investigation of the callose deposition pattern during megasporogenesis in *S. sediforme* and *S. rupestre* confirmed our previous assumptions that the thickened walls observed between the dyad and triad cells on the electronograms are related to callose deposition in these areas (Brzezicka and Kozieradzka-Kiszkurno 2019, 2021). The findings of the present study showed the relationship between the callose deposition pattern and the ultrastructure of the megaspores. Moreover, aniline blue staining identified callose in the side walls of the cells formed during megasporogenesis in *Sedum* ser. *Rupestria*. However, callose accumulation in megaspores of *S. hispanicum* is primarily limited to the transverse walls separating the individual cells of the dyad and linear tetrad similar to the majority of cases described in angiosperms (Rodkiewicz 1970; Tucker and Koltunow 2009; Rojek et al. 2018).

So far, the presence of the characteristic feature for Crassulaceae plasmodesmata has been observed only in *S. hispanicum* during megasporogenesis. We speculate that callose accumulation in the cell walls (Rodkiewicz 1970) and the presence of the electron-dense material near the plasmodesmata (Wróbel-Marek et al. 2017) affect the transport of substances between cells during embryological development. It has been suggested that the decrease in the cell wall permeability via callose deposition makes it a temporal selective barrier that blocks the translocation of specific signals that are crucial for meiotic division (Rodkiewicz 1970; Bouman 1984). Moreover, callose deposition is observed in the cell walls of higher plants and also at plasmodesmata. Callose regulates the communication between cells, including the movement of nutrients and molecules via plasmodesmata (Chen and Kim 2009; Tucker and Koltunow 2014; Azim and Burch-Smith 2020). The characteristic structure of *S. hispanicum* plasmodesmata and the different pattern of callose deposition (compared with other *Sedum* and also the general pattern studied so far—the presence of hypostasis with callose) have, in our opinion, the potential for providing a deeper understanding of intercellular communication between sporophyte and gametophyte tissues in the ovules of sexual species. Studies carried out during embryogenesis of the Crassulaceae species have indicated that plasmodesmata with an electron-dense material are functional (Wróbel-Marek et al. 2017). However, there are also reports that the symplasmic transport with their participation can be regulated or even unidirectional. This observation is the basis for the relationship between the presence of plasmodesmata with the adjacent electron-dense material and the rapid disappearance of callose during megasporogenesis in *Sedum*. To further understand the function of plasmodesmata and the direction of the transport during megasporogenesis, in-depth studies taking into account the new observation of the transport of substances between the cells are required. In the present study, aniline blue staining suggests that callose is finally absent in the walls of the megaspores at the tetrad stage and the FM when plasmodesmata with electron-dense material occur. This observation supports the current working hypothesis that callose is not a part of the electron-dense material located near the plasmodesmata formed during megasporogenesis, so probably it is not a callose plug. This observation is consistent with that of the recent immunolocalization studies conducted in our laboratory (Kozieradzka-Kiszkurno, unpubl.).

## Conclusion

The findings of the present study supplement the embryological understanding of the three *Sedum* species studied. Based on callose detection during megasporogenesis, it can be concluded that this cell wall polysaccharide isolates reproductive cells from the surrounding ovule somatic cells and that the callose deposition pattern is related to FM selection also in *Sedum*. These results reveal new, although quite discreet, cytoembriological characteristics of the studied plants, which confirm the similarity of embryological features among *Sedum* ser. *Rupestria*, which distinguish them from *S. hispanicum*. The present study confirms the hypothesis that the callose deposition pattern differs between the representatives of the genus *Sedum*, which is also reflected in the systematic position of the species. Moreover, these results suggest that callose is absent in the structure of the electron-dense material present near plasmodesmata on the side of the megaspore cytoplasm during megasporogenesis in *S. hispanicum*. To understand the importance of plasmodesmata with the electron-dense material formed during embryological development in the representatives of the family Crassulaceae, further research on the symplasmic contact of female generative cells and sporophyte tissues is needed.
